# Antidepressant efficacy of low-frequency repetitive transcranial magnetic stimulation in antidepressant-nonresponding bipolar depression: a single-blind randomized sham-controlled trial

**DOI:** 10.1186/s40345-021-00245-1

**Published:** 2021-12-08

**Authors:** Arthur D. P. Mak, Sebastiaan F. W. Neggers, Owen N. W. Leung, Winnie C. W. Chu, Jenny Y. M. Ho, Idy W. Y. Chou, Sandra S. M. Chan, Linda C. W. Lam, Sing Lee

**Affiliations:** 1 Department of Psychiatry, The Chinese University of Hong Kong, G/F Multicentre, Tai Po Hospital, Tai Po, Hong Kong, SAR China; 2 Department of Psychiatry, Brain Center Rudolf Magnus, University Medical Center Utrecht, Utrecht, The Netherlands; 3 Department of Imaging and Interventional Radiology, The Chinese University of Hong Kong, Hong Kong, SAR China

**Keywords:** Bipolar disorder, Depression, Transcranial magnetic stimulation, Randomized controlled trial

## Abstract

**Background:**

To examine the antidepressant efficacy and response predictors of R-DLPFC-LF rTMS for antidepressant-nonresponding BD.

**Methods:**

We conducted a single-blind randomized sham-controlled trial for 54 (28 sham, 26 active) patients with antidepressant-nonresponding BD (baseline MADRS ≥ 20). Patients received 15 daily sessions of active or sham neuronavigated rTMS (Figure-of-8 coil, five 1 Hz 60 s 110% RMT trains). Outcome measures included depressive response (≥ 50% MADRS reduction, CGI ≤ 2) and remission (MADRS < 7, CGI = 1) rates, treatment emergent hypo/mania (YMRS), depressive and anxiety symptoms (HAM-A).

**Results:**

48 patients (25 sham, 23 active) completed treatment, with 3 drop-outs each in active and sham groups. Active rTMS did not produce superior response or remission rates at endpoint or 6 or 12 weeks (ps > 0.05). There was no significant group * time interaction (ps > 0.05) in a multivariate ANOVA with MADRS, HAMA and YMRS as dependent variables. Exploratory analysis found MADRS improvement to be moderated by baseline anxiety (p = 0.02) and melancholia (p = 0.03) at week 3, and depressive onset at weeks 6 (p = 0.03) and 12 (p = 0.04). In subjects with below-mean anxiety (HAMA < 20.7, n = 24), MADRS improvement from active rTMS was superior to sham at week 3 (ITT, t = 2.49, p = 0.04, Cohen’s d = 1.05). No seizures were observed. Groups did not differ in treatment-emergent hypomania (p = 0.1).

**Limitations:**

Larger sample size might be needed to power subgroup analyses. Moderation analyses were exploratory. Single-blind design. Unblinding before follow-up assessments due to ethical reasons.

**Conclusions:**

1-Hz 110% RMT (5 × 60 s trains) **R**-DLPFC-LF rTMS was not effective for antidepressant non-responding BD but may be further investigated at increased dosage and/or in BD patients with low anxiety.

*Trial registration* CCRB Clinical Trials Registry, CUHK, CUHK_CCT00440. Registered 04 December 2014, https://www2.ccrb.cuhk.edu.hk/registry/public/279

**Supplementary Information:**

The online version contains supplementary material available at 10.1186/s40345-021-00245-1.

## Background

Depressive episodes dominate the illness course of Bipolar Disorder (Judd et al. 2005), which is a disabling condition with significant suicide risk that affects approximately 4% of the population (Kessler et al. 2005; Zarate et al. 2000; Goldstein et al. 2012). Treatment resistance is also common in bipolar depression—40% for 8 weeks of quetiapine and even less favorable in other first-line options such as lithium lamotrigine, olanzapine or olanzapine–fluoxetine combination (De Fruyt et al. 2012; Sidor and Macqueen 2011; Geddes et al. 2009), and unlike for unipolar depression, antidepressants commonly lead to non-response, rapid cycling and precipitating manic switches (Fela-Thomas et al. 2018; Viktorin et al. 2014).

Repetitive transcranial magnetic stimulation (rTMS) has recently gained recognition as an effective treatment for bipolar depression, with left-DLPFC high frequency (L-DLPFC-HF) rTMS obtaining FDA approval for bipolar depression (Neuronetics 2020). Earlier research, however, suggested that right-DLPFC low frequency (R-DLPFC-LF) rTMS may have comparable antidepressant efficacy for Major Depressive Disorder (Chen et al. 2013; Cao et al. 2018; Dell'Osso et al. 2015; Eche et al. 2012), but obtainable in shorter treatment time (4–9 min versus 30 min in L-HF) (Eche et al. 2012; Dell'Osso et al. 2009), with reduced discomfort at site of stimulation (Kaur et al. 2019) and a practically non-existent risk of seizure-induction (Sun et al. 2012; Theodore et al. 2002). Antidepressant efficacy of R-DLPFC-LF rTMS for bipolar depression has only received preliminary support mainly from open-label and active comparison non-inferiority trials, (Dell'Osso et al. 2009, 2015; Pallanti et al. 2014; Fitzgerald et al. 2006; Dell’osso and Altamura 2009). The only randomized sham-controlled trial (RCT) done so far, failed to demonstrate significant benefit of R-DLPFC-LF or L-DLPFC-HF rTMS over sham control (Hu et al. 2016) possibly attributable to a small sample size (L-HF on 12 subjects, R-LF on 13 subjects and 13 receiving sham). The effectiveness of R-DLPFC-LF rTMS for bipolar depression therefore needs to be examined in a larger sample. On the other hand, response to rTMS in depressive states is heterogeneous (Fitzgerald et al. 2016), with widely varied response rates from 42 to 75% (Dell'Osso et al. 2009; Pallanti et al. 2014; Fitzgerald et al. 2006; Hu et al. 2016; Kazemi et al. 2016), influenced by clinical factors such as depressive severity (Fitzgerald et al. 2016; Trevizol et al. 2002), anxiety (Trevizol et al. 2002; Brakemeier et al. 2007), medication use (Harel et al. 2011), and course variables (Fitzgerald et al. 2016). It would therefore be of interest to examine determinants of response, and identify potential subgroups of bipolar depressed patients that would show a superior response to R-DLFPC-LF rTMS.

In view of the above, we conducted a randomized single-blind sham-controlled study that examined the antidepressant efficacy of 3-week augmentative neuronavigated 1-Hz R-DLFPC-LF rTMS in 64 adults with antidepressant non-responding bipolar depression. We also performed moderation analysis to examine the influence of baseline clinical parameters, including depressive (Fitzgerald et al. 2016; Trevizol et al. 2002) and anxiety severity (Trevizol et al. 2002; Brakemeier et al. 2007), medication use (Harel et al. 2011), melancholic vs. atypical features, and course variables (Fitzgerald et al. 2016), on clinical response to the rTMS regime.

## Methods

### Study design

This study is a randomized, sham-controlled, single-blind trial of 3-week augmentative neuro-navigated 1-Hz R-DLPFC rTMS for antidepressant non-responding bipolar depression with post-treatment follow up assessments up to 12 weeks from treatment endpoint.

### Subjects

We recruited and randomized 64 right-handed patients aged 18 to 65 who met DSM-5 criteria for bipolar I or II disorder with a current major depressive episode (MDE) that showed no response to at least one previous adequate antidepressant trial (defined as having ≤ 25% reduction of MADRS following full or best tolerated dose of an antidepressant drug [bupropion or SSRI apart from paroxetine, commensurate with 3rd-line treatment recommendations in CANMAT 2013 guidelines (Yatham et al. 2013) for at least 6 weeks] in addition to at least one mood stabilizer (lithium, sodium valproate or lamotrigine). Only subjects with at least moderately severe depressive symptoms (Montgomery-Åsberg Depression Rating Scale [MADRS] ≥ 20) at treatment commencement were included in the analysis. The patients and their responsible psychiatrists were advised to avoid alterations to psychiatric medication and report whenever such alterations were necessitated. Patients with organic brain syndromes, current psychotic symptoms, mental retardation, substance use in recent 3 months, suicidal ideation or attempt in past month, obsessive–compulsive disorder, post-traumatic stress disorders, eating disorders, metallic implants, current pregnancy, unstable cardiac disease, personal or known 1st degree relatives’ history of seizures were excluded. None of the participants had previously received rTMS nor had record of non-response to electroconvulsive therapy.

### Randomization and blinding

64 subjects were randomly allocated to the active intervention or sham control group on a 1:1 ratio using a random allocation sequence obtained from a computer-generated list of random numbers in blocks of 10 by a statistician with no other involvement in the study. The allocation was concealed from patients and the researchers responsible for data collection and analysis, but not the TMS therapists. The TMS therapists was forbidden from disclosing the randomized allocation status to the participants. Participants were unblinded on the day of their final treatment session after progress evaluation. Those in the sham control group were offered the option of receiving active TMS treatment, where recommended by their responsible psychiatrists, after treatment unblinding. Where open-label active TMS treatment commenced within the follow-up assessment period, all subsequent assessment data were excluded in per-protocol (PP) analysis and imputed with last observation carried forward method in intention-to-treat (ITT) analysis.

### Neuronavigation

Prior to treatment, subjects underwent a structural brain MRI using a Philips 3.0-T whole-body scanner (Achieva TX, Philips Healthcare, Best, the Netherlands). Whole brain anatomical datasets were acquired with a T1-weighted sequence (repetition time (TR)/echo time (TE): 7.6/3.5 ms, field of view 230 mm, 250 contiguous slices, 0.6 mm thickness, reconstruction matrix 224 × 224). The datasets were installed on a neuro-navigation software (Brainsight 2 Neuronavigator for TMS, Rogue Research Inc. 2007) to guide coil placement over the right dorsolateral prefrontal cortex (RDLPFC) (MNI X, Y, Z [SD] = 35.71 [5.81], 44.63 [8.72], 31 [8.08]), corresponding to areas between 9 and 46 (middle third of the middle frontal gyrus and most rostral portion of inferior frontal gyrus) in the original Brodmann Map.

### TMS parameters

70 mm Magstim figure-of-eight coil was hand-held in place guided by neuronavigation, tangential to the scalp with the handle pointing back and away from the midline at 45°.

TMS treatment were delivered using Magstim Super Rapid 2 with a 70 mm figure-of-eight coil. The coil was held tangential to the scalp with the handle pointing back and away from the midline at 45°. Treatment parameters were based on Dell’Osso’s single-arm study (Dell'Osso et al. 2009). During each session, subjects were given 300 pulses of stimulation at 1 Hz divided into 5 trains of 60 stimuli, each train separated from the next by a one-minute pause. Subjects received 15 sessions over weekdays of 3 consecutive weeks, with a total of 4500 stimuli over the full intervention course. rTMS was delivered at 110% of subjects’ resting motor threshold, defined as the minimum magnetic intensity required to elicit 5 motor evoked potentials (50 μV), as measured using a Brainsight EMG amplifier, out of 10 consecutive stimuli in the abductor pollicis brevis.

Neuronavigation, positioning, protocol of treatment for the sham treatment group, including weekly determination of resting motor threshold, were identical to that in the treatment group, except that sham treatment was delivered with a Magstim sham coil, essentially an inactive figure-of-eight coil which looked identical and produced sounds mimicking the frequency and loudness of the active coil.

### Diagnostic and symptomatic evaluation

Diagnoses were obtained using the validated Chinese-bilingual version of the Structured Clinical Interview for DSM Mental Disorders (SCID–C/B) (So et al. 2003) by trained research assistants under supervision of an experienced academic psychiatrist (AM). The severity of depressive, anxiety and manic symptoms were evaluated respectively using Montgomery-Åsberg Depression Rating Scale (MADRS) (Montgomery and Asberg 1979), Hamilton Anxiety Rating Scale (HAMA) (Hamilton 1958) and Young Mania Rating Scale (YMRS) (Young et al. 1978). Subjects were also evaluated using Clinical Global Impression Scale (CGI) (Guy 1976) to reflect global clinical severity. Symptom severity was evaluated before the first treatment session (week 0), after the last treatment session (week 3), and at two post-treatment follow-ups three (week 6) and 6 weeks (week 12) after the last treatment session.

### Data analysis

In the 64 randomised subjects, 6 allocated to the active arm and 4 in the sham arm had MADRS score dropped below 20 by treatment commencement and became ineligible. Intention-to-treat analysis included the 54 participants with MADRS ≥ 20 and entered randomized treatment phase (26 active rTMS, 28 sham rTMS). Missing data (treatment termination, follow-up assessments conducted after the start of open-label active treatment, interview decline) were removed in per protocol analysis (see Additional file 1: Fig. S1), and imputed with last observation carried forward in ITT analysis (see Additional file 2: Fig. S2).

The primary outcome measure was clinical response (defined as ≥ 50% reduction in MADRS score and CGI ≤ 2) in depressive symptoms at treatment endpoint. Secondary outcome measures included changes in depressive, anxiety and manic symptom severity, endpoint remission rate (defined as MADRS < 7 and CGI = 1), sustained response and remission rates at weeks 6 and 12, and treatment-emergent hypomanic or manic episodes by treatment endpoint.

Differences in response and remission rates were compared between the active intervention and sham control groups were using the Fisher’s exact test.

Multivariate repeated measures ANOVA was used as a global test to identify any between-group differences in depressive, anxiety and hypomanic symptoms over time while limiting the joint error rate. Univariate ANOVAs were also performed separately for depressive, anxiety and hypomanic symptoms to gauge their respective effect sizes. Time contrasts (each assessment timepoint contrasted with the subsequent) were also applied to identify when group differences emerge.

As an explorative analysis, moderation analysis was performed to identify if antidepressant effect depended upon demographic (age, sex, education level) and clinical variables (baseline MADRS, HAMA, YMRS, bipolar subtype (I vs. II), age of first depressive onset, years since depressive onset, length of current depressive episode, melancholic, atypical and rapid cycling specifier lifetime and current number of comorbid mental disorders) at each of the timepoints. The moderation effects of antidepressants and antipsychotics use at baseline were also tested on MADRS change at week 3, but not at follow-up assessments in the absence of data on post-treatment medication changes. As the explorative moderation analysis is aimed at identifying potential response predictors from a broad range of 16 candidate variables, correction for multiple comparison was not applied at this stage.

Significant moderators were then used to stratify the sample (with/without the characteristic for binary variables, above/below average for continuous variables). In each of the resultant subgroups, Holms–Bonferroni corrected t-tests were conducted to compare the level of MADRS improvement in active versus sham treatment.

All statistical analyses were conducted with Python 3.8. Results with two-sided p values lower than 0.05 were considered significant.

## Results

26 subjects from active and 28 subjects from sham treatment groups were included in ITT analysis. Subjects were on average 40 (SD 11.45) years old, 67% were female, over half (59%) received post-secondary education. Most of the subjects were of bipolar II (85.2%, n = 46 vs. bipolar I 14.8%, n = 8), with no significant difference between treatment and sham groups (p = 1.0). All subjects were at least moderately depressed (MADRS ≥ 20). The two groups did not differ in baseline psychopharmacological profile or any demographic, symptom or course variables (p > 0.05) (see Table 1).

**Table 1:** Demographic and clinical variables

	ITT				PP			
	Sham	Treatment	t/chi^2^	p	Sham	Treatment	t/chi^2^	p
	n = 28	n = 26			n = 25	n = 23		
Age, mean (SD)	39.4 (11.3)	40.7 (11.4)	− 0.4	0.69	40.0 (11.4)	39.7 (11.6)	0.09	0.93
Sex, male n (%)	10 (36)	8 (31)	0.01	0.92	9 (36)	8 (35)	0.05	0.83
Education, n (%)			–	0.70			–	0.5
Primary or below	1 (4)	1 (4)			1 (4)	1 (4)		
Secondary	12 (43)	8 (31)			12 (48)	7 (30)		
Post-secondary	15 (54)	17 (65)			12 (48)	15 (65)		
Bipolar disorder subtype, n (%)			–	1			–	1
Type 1	4 (14)	4(15)			4 (16)	4 (17)		
Type 2	24 (86)	22 (85)			21 (84)	19 (83)		
Baseline MADRS, mean (SD)	27.0 (5.1)	27.5 (5.2)	− 0.41	0.68	26.2 (3.8)	26.8 (4.9)	− 0.48	0.63
Baseline HAM-A, mean (SD)	20.5 (7.0)	21.0 (10.3)	− 0.19	0.85	21.0 (7.1)	20.0 (10.4)	0.38	0.71
Baseline YMRS mean (SD)	1.0 (1.9)	2.3 (3.2)	− 1.83	0.07	1.1 (2.0)	1.7 (2.4)	− 0.82	0.42
Melancholic specifier, n (%)	18 (64)	17 (65)	0.04	0.84	16 (64)	14 (61)	0.01	0.94
Atypical specifier, n (%)	6 (21)	13 (50)	3.65	0.06	6 (24)	11 (48)	2.02	0.16
Rapid cycling specifier, n (%)	13 (46)	17 (65)	1.27	0.26	12 (48)	15 (65)	0.83	0.36
Onset	23.5 (8.4)	23.9 (9.0)	− 0.18	0.86	23.7 (8.5)	23.7 (8.8)	− 0.02	0.98
Years since onset	15.9 (8.9)	17.6 (9.5)	− 0.69	0.5	16.3 (8.8)	17.0 (9.7)	− 0.23	0.82
On antidepressant at baseline, n (%)	19 (68)	19 (73)	0.01	0.9	17 (68)	17 (74)	0.02	0.9
On antipsychotic at baseline, n (%)	24 (86)	20 (77)	–	0.49	22 (88)	19 (83)		0.7
Number of comorbid disorders (lifetime)	1.9 (1.3)	2.3 (1.2)	− 0.99	0.33	2.0 (1.3)	2.3 (1.2)	− 0.83	0.41
(Current)	1.5 (0.9)	2.0 (1.1)	− 1.85	0.07	1.6 (0.9)	2.0 (1.1)	− 1.52	0.14

*ITT* intention-to-treat, *PP* per-protocol, *MADRS* Montgomery–Åsberg Depression Rating Scale, *HAMA* Hamilton Anxiety Rating Scale, *YMRS* Young Mania Rating Scale

23 and 25 subjects in the active and sham treatment arms, respectively, completed 3 weeks of study treatment. Treatment was terminated for 3 subjects from each group in the first week of study treatment. 2 subjects from the active group were terminated due to erratic drug use, and one reported suicidal ideation that was not disclosed prior to recruitment screening, and was then referred for emergency clinical care. Two subjects from the sham group withdrew citing scheduling difficulties and one withdrew stating intention to seek alternative treatment.

### Main results

In multivariate ANOVAs with MADRS, HAMA and YMRS as dependent variables, time × group effects were insignificant for both ITT (F[9, 44] = 1.36, p = 0.23, ηp2 = 0.22) and PP (F[9, 31] = 1.52, p = 0.19, η_p_^2^ = 0.31) analyses (see Table 3).

**Fig. 1:**
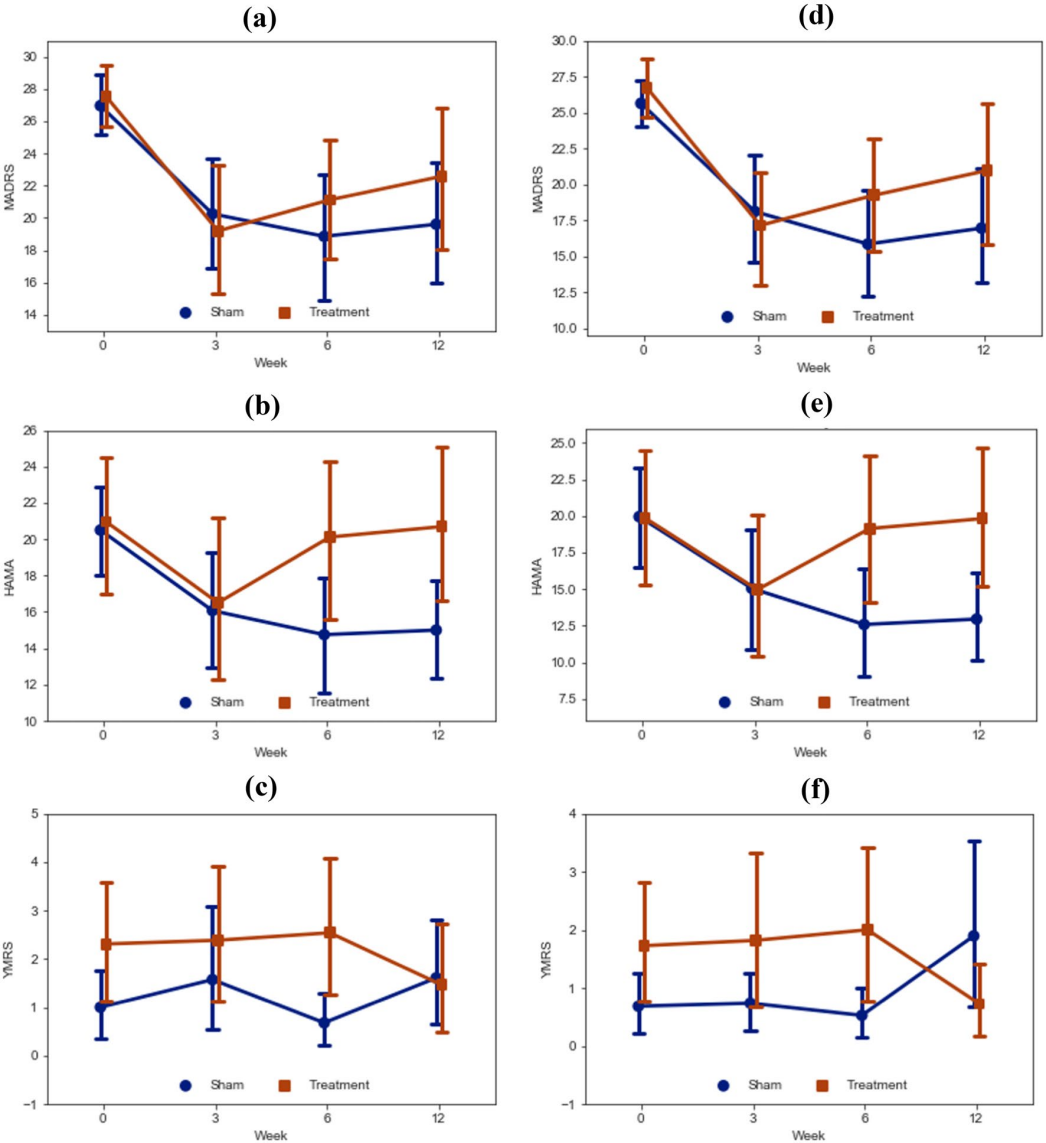
Marginal means of MADRS, YMRS and HAMA across assessment timepoints. *MADRS* Montgomery–Åsberg Depression Rating Scale, *HAMA* Hamilton Anxiety Rating Scale, *YMRS* Young Mania Rating Scale. **a**–**c** Intention-to-treat. **d**–**f** Per-protocol

In univariate MADRS analysis, the time × group effect was insignificant and with small effect size (ITT: F[3, 156] = 1.07, p = 0.37, ηp2 = 0.02, PP: F[3, 117] = 1.1, p = 0.35, ηp2 = 0.03) (see Table 3). The only timepoint where MADRS improvement from baseline was higher in active group (ITT 8.34 [SD 6.71], PP 9.43 [7.63]) than in sham (ITT 6.71 [8.28], PP 7.52 [8.41]) was week 3 (see Fig. 1), but the group * time effect was insignificant (Baseline—week 3 contrast ITT: F[1, 52] = 0.56, p = 0.46, ηp2 = 0.01 PP: F[1, 39] = 0.61, p = 0.44, ηp2 = 0.02) (see Table 3).

### Response and remission

Response rates did not differ significantly between the two groups at week 3 (ITT—active 12% vs. sham 11%; PP—active 13% vs. sham 12%) or at week 6 or 12 follow ups (ITT—active 8% vs. sham 7%; PP—active 9% vs. sham 9–11%). One subject receiving active intervention (ITT 4%, PP 4%) and none from sham group remitted at week 3. Remission was sustained upon week 12 (active vs. sham p > 0.05) (see Table 2).

**Table 2:** Fisher’s exact test of response and remission rates and treatment-emergent manic/hypomanic episodes

	ITT^a^			PP^b^		
	Sham	Active	p	Sham	Active	p
Response^c^						
Week 3	3 (11)	3 (12)	1	3 (12)	3 (13)	1
Sustained at week 6	2 (7)	2 (8)	1	2 (9)	2 (9)	1
Sustained at week 12	2 (7)	2 (8)	1	2 (11)	2 (9)	1
Remission^d^						
Week 3	0 (0)	1 (4)	0.48	0 (0)	1 (4)	0.48
Sustained at week 6	0 (0)	1 (4)	0.48	0 (0)	1 (4)	1
Sustained at week 12	0 (0)	1 (4)	0.48	0 (0)	1 (5)	1
Manic or hypomanic episodes by week 4	3 (11)	8 (31)	0.095	3 (12)	7 (30)	0.16

^a^Sham n = 28, treatment n = 26

^b^Sham n at week 3, 6 and 12 = 25, 23, 19; treatment n at week 3, 6 and 12 = 23, 23, 22

^c^Response defined as 50% reduction in MADRS from baseline and CGI ≤ 2

^d^Remission defined as MADRS < 7 and CGI = 1

### Exploratory moderation analyses and subgroup analyses

**Fig. 2:**
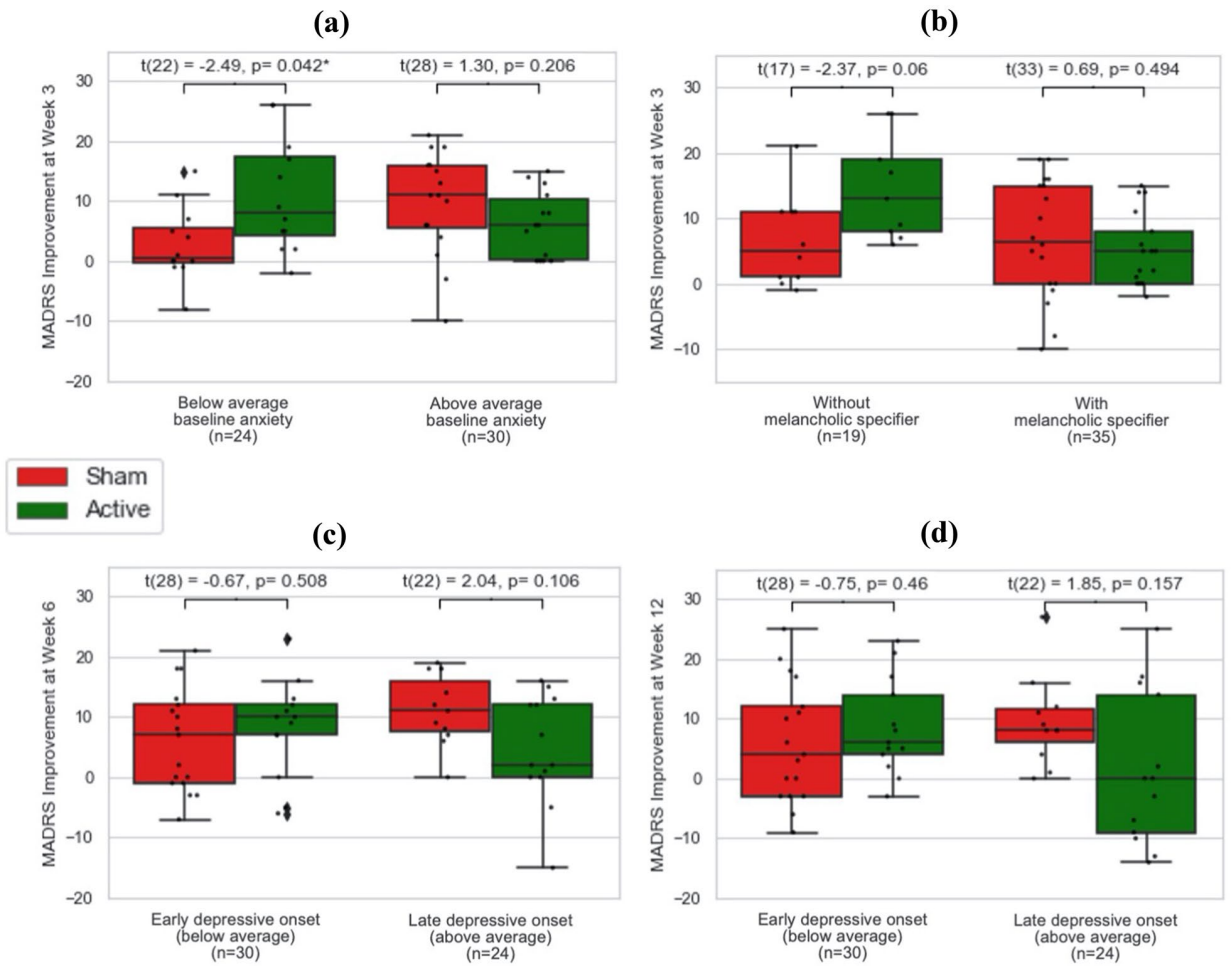
Variables interacting with MADRS improvements in intention-to-treat analysis. MADRS, Montgomery–Åsberg Depression Rating Scale; *p < 0.05. **a** Baseline anxiety * treatment group interaction at week 3: β = − 0.62 95% CI (− 1.14–0.10), p = 0.02. **b** Melancholic specifier * group interaction at week 3: β = − 9.83 95% CI (− 18.46–1.20), p = 0.03. **c** Onset * group interaction at week 6: β = − 0.58 95% CI (− 1.09–0.07), p = 0.03. **d** Onset * group interaction at week 12: β = − 0.66 95% CI (− 1.29–0.03), p = 0.04. *Significant at p < 0.05

Exploratory moderation analysis showed that week 3 improvement in depression (MADRS) was moderated by baseline anxiety (interaction p = 0.02) (see Fig. 2a) and melancholic features (interaction p = 0.03) (see Fig. 2b). No significant moderation effect on week 3 depressive symptoms was found from other clinical variables such as baseline antidepressant use, bipolar subtype or age of depressive onset. Subgroup analysis then proceeded with subjects stratified by baseline anxiety score, and found significantly greater MADRS improvement in active compared to sham group in those with below-mean (HAM-A = 20.7, n = 24) baseline anxiety (t = − 2.49, Holms-Bonferroni corrected p = 0.04, Cohen’s d = 1.05) (see Fig. 2a). No significant active-sham difference in MADRS change was found in the sub-groups stratified by baseline melancholic features (see Fig. 2b).

**Fig. 3:**
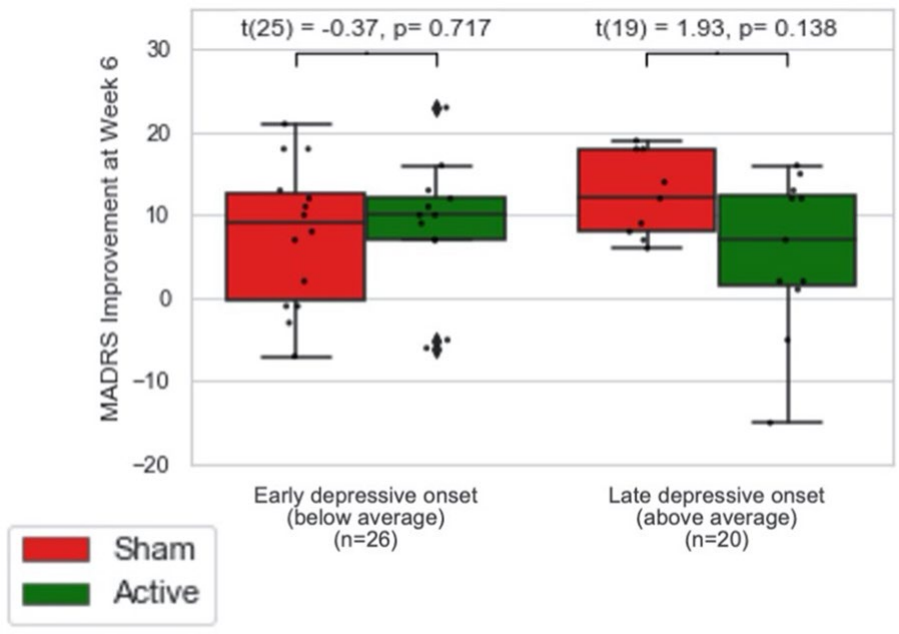
Variables interacting with MADRS improvements in per-protocol analysis. *MADRS* Montgomery–Åsberg Depression Rating Scale. Onset * group interaction: β = − 0.66 95% CI (− 1.28–0.04), p = 0.04

At week 6 and 12, age of depressive onset (week 6 and 12 interaction ps = 0.03, 0.04) (see Fig. 2c, d) moderated MADRS improvement in ITT analysis. This moderation effect was also significant in PP analysis at week 6 but not 12 (interaction p = 0.04) (see Fig. 3). None of the subgroup analyses stratified by age of onset showed significant group * time interaction at week 6 or 12. No significant moderation effect on week 6 or 12 depressive symptoms were found with any other clinical variables.

### Secondary outcomes—anxiety and manic symptoms

There were weak time * group interactions for anxiety (ITT: F[3, 156] = 2.8, p = 0.04, ηp2 = 0.05; PP: F[3, 117] = 3.14, p = 0.03, ηp2 = 0.08) and hypomanic symptoms (PP: F[3, 117] = 3.39, p = 0.02, ηp2 = 0.08) in univariate ANOVA that were not significant in the multivariate analysis (see Table 3).

**Table 3:** ANOVA time * group interaction effects

		ITT				PP			
		F	df	Sig	ηp2	F	df	Sig	ηp2
ANOVA across all time points									
Multivariate									
MADRS, HAMA, YMRS		1.36	9, 44	0.23	0.22	1.52	9, 31	0.19	0.31
Univariate									
MADRS		1.07	3, 43.77	0.37	0.02	1.1	3, 52.75	0.35	0.03
HAMA		2.8	3, 116.39	0.04*	0.05	3.14	3, 157.54	0.03*	0.08
YMRS		1.99	2.57, 11.41	0.13^a^	0.04	3.39	3, 14.66	0.02*	0.08
Time contrasts (each time point contrasted to the subsequent)									
MADRS									
Baseline vs. week 3		0.56	1, 52	0.46	0.01	0.61	1, 39	0.44	0.02
Week 3 vs. week 6		1.8	1, 52	0.19	0.03	2.02	1, 39	0.16	0.05
Week 6 vs. week 12		0.09	1, 52	0.77	0	0.04	1, 39	0.85	0
HAMA									
Baseline vs. week 3		0	1, 52	0.99	0	0	1, 39	1	0
Week 3 vs. week 6		5.73	1, 52	0.02*	0.1	6.49	1, 39	0.02*	0.14
Week 6 vs. week 12		0.02	1, 52	0.89	0	0.01	1, 39	0.92	0
YMRS									
Baseline vs. week 3		0.32	1, 52	0.57	0.01	0	1, 39	0.96	0
Week 3 vs. week 6		1.09	1, 52	0.3	0.02	0.16	1, 39	0.7	0
Week 6 vs. week 12		6.41	1, 52	0.01*	0.11	6.56	1, 39	0.01*	0.14

*ITT* intention-to-treat, *PP* per-protocol, *MADRS* Montgomery–Åsberg Depression Rating Scale, *HAMA* Hamilton Anxiety Rating Scale, *YMRS* Young Mania Rating Scale

*p < 0.05

^a^Sphericity not assumed, Greenhouse–Geisser test used instead

The interaction in anxiety symptoms emerged after the end of treatment—whereas anxiety symptoms continued to decrease in sham, it rebounded in active treatment (week 3–6 contrast ITT: F[1, 52] = 5.73, p = 0.02, ηp2 = 0.1, PP: F[1, 39] = 6.49, p = 0.02, ηp2 = 0.14). No other contrasts were significant (see Table 3).

The interaction in hypomanic symptoms emerged between the two post-treatment follow-ups (week 6–12 contrast)—hypomanic symptoms decreased in active treatment group but increased in sham treatment group (ITT: F[1, 52] = 6.41, p = 0.01, ηp2 = 0.11; PP: F[1, 39] = 6.56, p = 0.01, ηp2 = 0.14). No other contrasts were significant (see Table 3).

There was no report of any treatment-emergent seizures or other serious adverse events. The rate of treatment-emergent hypomania did not differ significantly between active intervention (ITT 31%, PP30%) and sham control (ITT 11%, PP12%) groups, (Fisher’s exact test: ITT p = 0.1, PP p = 0.16) (Table 2) and active treatment was not associated with significantly increased YMRS between any time points (see Table 3).

## Discussion

In this randomized sham rTMS-controlled, single-blind study, we did not observe significant antidepressant effect from 1 Hz R-DLPFC rTMS for antidepressant-nonresponding bipolar depressed patients, but observed a significant antidepressant effect in those with low baseline anxiety.

The additional improvement in depressive symptoms in active versus in sham intervention at week 3 was small (ITT 1.64 point difference). The small effect size (ηp2 ITT = 0.01) means that a much larger sample (n = 134 per arm) would be required to detect a significant effect. This was far larger than the sample size used in the present study, which was estimated a priori based on single-arm data (55% response rate, versus 12–13% obtained in the present study), when no controlled comparison was available (Dell'Osso et al. 2009). The negative results from another, even smaller randomized controlled trial (n = 13 per arm) (Hu et al. 2016) of L-DLPFC or R-DLPFC rTMS on bipolar II depression (Hu et al. 2016) is therefore not surprising.

The response and remission rates were lower than those previously reported (42–75% response; 13–40% remission) (Dell'Osso et al. 2009; Pallanti et al. 2014; Fitzgerald et al. 2006; Hu et al. 2016; Kazemi et al. 2016). The discrepancy is unlikely attributable to differences in sample characteristics since this sample did not differ vastly in age, depressive severity or treatment resistance compared to those in previous studies (Dell'Osso et al. 2009; Pallanti et al. 2014; Fitzgerald et al. 2006; Hu et al. 2016; Kazemi et al. 2016). As expected by the study design for recruiting antidepressant-nonresponding bipolar depressed patients, a rather high proportion of (70%) patients in our sample received concurrent antidepressant treatment, whereas 23% (Kazemi et al. 2016) to 91% of patients from previous R-DLPFC-LF rTMS studies (Dell'Osso et al. 2009) received antidepressant treatment. In lieu of clear evidence on the impact of concurrent antidepressant drug use on rTMS effectiveness, it is difficult to assess if this was relevant in explaining the low response and remission rates found in this study. Instead, the discrepancies may reflect the more stringent response and remission criteria with the addition of CGI improvements in addition to changes in depressive symptom score (Dell'Osso et al. 2009; Pallanti et al. 2014; Fitzgerald et al. 2006; Hu et al. 2016; Kazemi et al. 2016), and the lower stimulation dosage compared to new stimulation protocols that have been found to have good effectiveness and tolerability profiles since this study has started—eg., with 5 additional rTMS sessions, or more pulses per session (total 420–1500), and shorter wait time between trains (Pallanti et al. 2014; Hu et al. 2016; Kazemi et al. 2016). Future studies should examine the effectiveness of R-DLPFC-LF rTMS regimes with either higher stimulation intensity, number of pulse per sessions or total number of sessions.

Nonetheless, we did observe a significant antidepressant effect from R-DLPFC-LF rTMS in low-anxiety bipolar depressed patients. Greater antidepressant effect in patients with low anxiety has been observed for L-DLPFC-HF rTMS (Trevizol et al. 2002; Brakemeier et al. 2007) and antidepressant drug (Fava et al. 2008; Saghafi et al. 2007). Effect size (Cohen’s d = 10.5) was large, and the level of improvement (mean 10.8 point MADRS reduction) appeared clinically meaningful, especially given previous non-response to antidepressant drugs which was required for enrolment in this study. The results at hand suggest that the present protocol could be beneficial to bipolar depressed patients with mild (HAM-A ≤ 17) and potentially some of the patients with mild to moderate (HAM-A 18–24) anxiety (Hamilton 1958). The insignificant moderation results at week 6 and 12 may either suggest that baseline anxiety did not influence maintenance of effect from rTMS, or the lack of sustained treatment effect from the current regime. Future studies exploring R-DLPFC-LF rTMS may consider increasing number of treatment sessions, treatment strength, or incorporating maintenance rTMS (Richieri et al. 2013).

In contrast to previous rTMS studies, response to R-DLPFC-LF rTMS in the present study was not influenced by baseline depressive severity or length of current episode (Fitzgerald et al. 2016). This could be related to this narrower range of depressive severity resulting from the exclusion of actively suicidal or psychotic patients who were likely more depressed (Melhem et al. 2019; Gaudiano et al. 2009). In Hong Kong, these patients would have been treated in in-patient settings where rTMS is unavailable. We observed non-melancholic depression to predict greater acute improvement in depressive symptoms, which stood in contrast to clinical trials of antidepressant drugs and electroconvulsive therapy where melancholic depression predicted superior response (Brown 2007). Previous rTMS studies have identified individual symptoms, cognitive vs. somatic symptoms, guilt, severity of sleep disruption and somatic anxiety as predictors of rTMS response (Poleszczyk et al. 2018), but few examined the effect of melancholic vs. atypical subtypes of depression. It has also remained unclear if certain common features of melancholia or atypicality, such as vegetative (or reversed) symptoms or psychomotor retardation would predict rTMS response in the way they would for ECT and antidepressant treatment. The effect of, and changes in these clinical phenomena should be further examined with more refined measures in future rTMS studies, incorporating imaging and electro-physiological investigations to examine the neurobiological mechanisms of these treatment modalities.

Nonetheless, we found R-DLPFC-LF rTMS to be safe, as reported in previous studies (Dell'Osso et al. 2009; Pallanti et al. 2014; Fitzgerald et al. 2006; Hu et al. 2016). There was no report of seizures or significant increase in manic symptoms or episodes.

Our study has several limitations. Firstly, a larger sample size may have been required to not only reveal between-group effects, but also adequately power identification of predictors and subsequent subgroup analyses. In fact, even though a stringent correction method was applied when splitting the sample for subgroup analysis, the moderation analyses used to highlight likely response predictors were itself uncorrected, and their results should thus be considered preliminary, at most highlighting potential candidate predictors for further study. Secondly, although bipolar subtype was not found to moderate clinical response, it should be noted that bipolar I only constituted 14.8% of this sample. Since subjects were recruited irrespective of bipolar subtype, and that both subtypes are associated with similar extents of depressive morbidity (Judd et al. 2005), this may reflect the more common occurrence of bipolar II than bipolar I (Merikangas et al. 2007) especially in specialist clinic settings (Akiskal et al. 2000). Nonetheless, findings in this study may have limited generalizability to bipolar I patients, and the association of bipolar subtype and response to R-DLPFC-LF rTMS should be examined in further studies. Thirdly, inherent limitations of the rTMS system used (Magstim) necessitated a single-blind design, which may not have the fidelity of allocation concealment of a double-blind design. Fourthly, although patients were explicitly advised to maintain their psychotropic regime throughout the rTMS trial, record of drug adherence required self-report and some patients may still have reduced medication dosage after experiencing antidepressant effects from rTMS, as various factors, including subjective recovery or improvement has been reported as reasons for treatment non-adherence (Arvilommi et al. 2014; Montes et al. 2013). Lastly, unblinding at week 3 may have influenced subsequent adjustments to psychotropic regimes with impact on mood change. Although we lack data to substantiate this speculation, it is plausible that more subjects from the sham group may have begun antidepressant drug after unblinding, which would be consistent with the sham group’s more sustained improvements at week 6–12 in antidepressant moderation analysis.

## Conclusions

R-DLPFC-LF rTMS was found to have a good safety and tolerability profile, but did not significantly reduce depressive symptoms in antidepressant-nonresponding bipolar depressed patients in general at the current dosage, although our preliminary findings suggested potential effectiveness in patients with low anxiety. Further sufficiently powered studies will be needed to examine if R-DLPFC-LF rTMS at a higher dosage is effective for bipolar depression, especially with low anxiety, where it may then become a viable alternative to conventional L-DLPFC-HF rTMS given the shorter treatment time and reduced discomfort.

## Supplementary Information

**Additional file 1: Figure S1.** Subject flowchart per protocol.

**Additional file 2: Figure S2.** Subject flowchart per intention-to-treat.

## Data Availability

The datasets used and/or analysed during the current study are available from the corresponding author on reasonable request.

## Abbreviations

BD: Bipolar depression
R-DLPFC-LF: Right-DLPFC low frequency
L-DLPFC-HF: Left-DLPFC high frequency
MADRS: Montgomery–Asberg Depression Rating Scale
YMRS: Young Mania Rating Scale
HAMA: Hamilton Anxiety Rating Scale
CGI: Clinical Global Impression Scale
PP: Per-protocol
ITT: Intention-to-treat
RDLPFC: Right dorsolateral prefrontal cortex
SCID: Structured Clinical Interview for DSM Mental Disorders
