# Supplementary figures and images for: Antidepressant efficacy of low-frequency repetitive transcranial magnetic stimulation in antidepressant-nonresponding bipolar depression: a single-blind randomized sham-controlled trial
Source: Int J Bipolar Disord. 2021 Dec 8;9:40. doi: 10.1186/s40345-021-00245-1 (PMC8651939; doi:10.1186/s40345-021-00245-1)

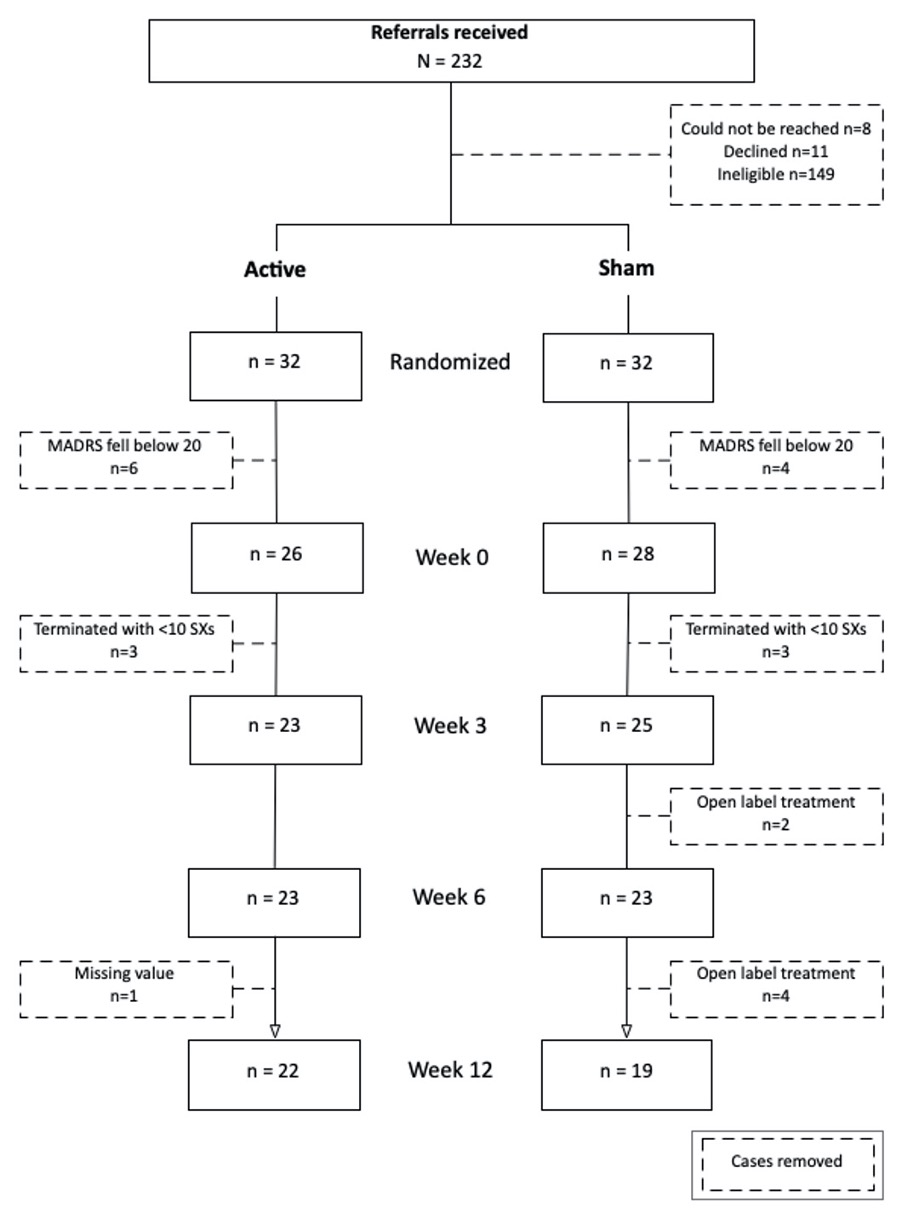

Supplement: Supplementary file 1 — Additional file 1: Figure S1. Subject flowchart per protocol. [file 40345_2021_245_MOESM1_ESM.jpg]

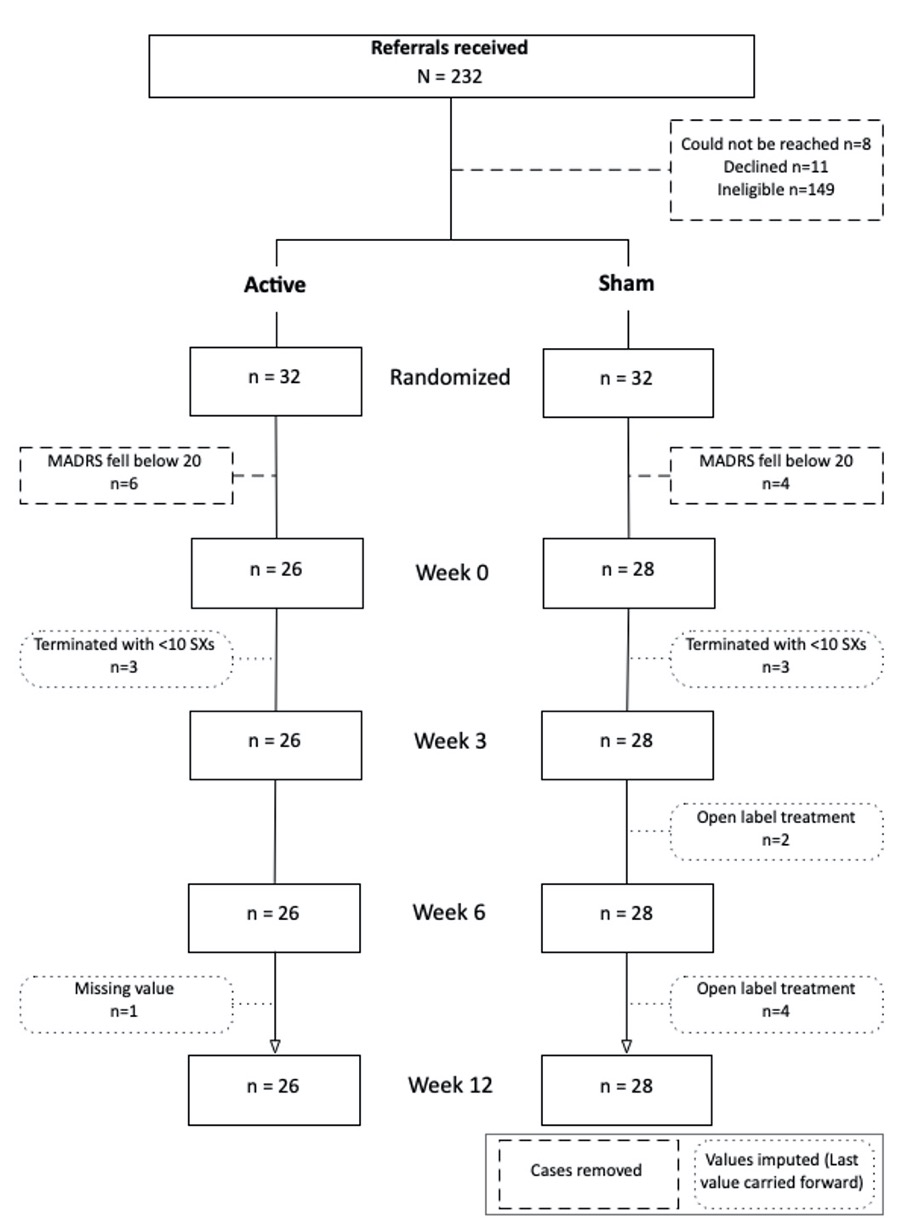

Supplement: Supplementary file 2 — Additional file 2: Figure S2. Subject flowchart per intention-to-treat. [file 40345_2021_245_MOESM2_ESM.jpg]
